# Cerebrospinal Fluid Presepsin As a Marker of Nosocomial Infections of the Central Nervous System: A Prospective Observational Study

**DOI:** 10.3389/fneur.2018.00058

**Published:** 2018-02-15

**Authors:** Sergey A. Abudeev, Kirill V. Kiselev, Nikolay M. Kruglyakov, Ksenia A. Belousova, Inna N. Lobanova, Oleg V. Parinov, Yuriy D. Udalov, Maxim A. Zabelin, Alexandr S. Samoilov, Evaldas Cesnulis, Tim Killeen, Konstantin A. Popugaev

**Affiliations:** ^1^Burnazian State Research Center, Federal Medical-Biological Agency, Moscow, Russia; ^2^Pirogov Russian National Research Medical University, Russian Ministry of Education, Moscow, Russia; ^3^Department of Neurosurgery, Klinik Hirslanden, Zurich, Switzerland

**Keywords:** nosocomial infection of the central nervous system, meningitis, ventriculitis, presepsin, inflammation

## Abstract

**Background:**

Nosocomial CNS infection (NI-CNS) is a common and serious complication in neurocritical care patients. Timely, accurate diagnosis of NI-CNS is crucial, yet current infection markers lack specificity and/or sensitivity. Presepsin (PSP) is a novel biomarker of macrophage activation. Its utility in NI-CNS has not been explored. We first determined the normal range of cerebrospinal fluid (CSF) PSP in a control group without brain injury before collecting data on CSF PSP levels in neurocritical care patients. Samples were analyzed in four groups defined by systemic and neurological infection status.

**Results:**

CSF PSP levels in 15 control patients without neurological injury were 50–100 pg/ml. Ninety-seven CSF samples were collected from 21 neurocritical care patients. In patients without NI-CNS or systemic infection, CSF PSP was 340.4 ± 201.1 pg/ml. Isolated NI-CNS was associated with CSF PSP levels of 640.8 ± 235.5 pg/ml, while levels in systemic infection without NI-CNS were 580.1 ± 329.7 pg/ml. Patients with both NI-CNS and systemic infection had CSF PSP levels of 1,047.7 ± 166.2 pg/ml. In neurocritical care patients without systemic infection, a cut-off value of 321 pg/ml gives sensitivity and specificity for NI-CNS of 100 and 58.3%, respectively.

**Conclusion:**

CSF PSP may prove useful in diagnosing NI-CNS, but its current utility is as an additional marker only.

## Introduction

Nosocomial infection of the central nervous system (NI-CNS) is a serious complication in neurocritical care patients, which leads to clinical deterioration, worsening of outcomes, and increased treatment costs (1–3). Risk factors include the duration of any neurosurgical intervention, perioperative cerebrospinal fluid (CSF) leakage, intraventricular hemorrhage, skull base fractures, prior antibiotic exposure, and the use of intracranial devices, especially if externalized (4, 5). Specifically, the implantation, duration of usage, irrigation, and disconnection of external ventricular drains represent important associated factors (4), with the risk of external ventricular drainage-related NI-CNS ranging from 10 to 27% (4, 6–8).

The timing of antibiotic administration in neurocritical care patients with suspected NI-CNS is of pivotal importance. Early antibiotic therapy improves outcomes, reduces intensive care unit (ICU) stay, and decreases treatment costs (9–11). Equally, unnecessary antibiotic use in neurocritical care patients without NI-CNS leads to eradication of resident flora and colonization with nosocomial bacteria, and may make subsequent central nervous system (CNS) infection more likely (12, 13).

Ideal antibiotic strategy can therefore only be achieved with accurate and rapid diagnosis of NI-CNS and pathogen verification. In many clinical situations, traditional diagnostic criteria are not specific. Alterations in consciousness and fever are common in neurocritical care patients and NI-CNS is one of a plethora of possible diagnoses. Meningism is typical of NI-CNS, but is also provoked by subarachnoid blood (4, 7, 14). Serum white cell counts, C-reactive protein (CRP), and procalcitonin (PCT) are invariably raised in patients with NI-CNS, but are also elevated in the acute phase of neurocritcal care pathologies and systemic infections (15, 16). Likewise, CSF cell count is elevated in NI-CNS and both subarachnoid and intraventricular hemorrhage (17). The presence of blood complicates the interpretation of CSF investigation; blood contains protein and cells, which consume glucose and secrete lactate to some degree, mimicking the effect of bacterial meningitis (18, 19). Even pathogen verification, by way of culture, polymerase chain reaction (PCR), or mass-spectrometry, does not always lead to a clear diagnosis of NI-CNS, as a judgment as to whether the results represent infection, contamination, or colonization must be made (11, 17, 20, 21).

Consequently, the search for new, reliable markers of NI-CNS is currently a focus of intensive research efforts. Recently, a new inflammation biomarker—presepsin (soluble CD14 subtype; PSP)—has been introduced into clinical practice (22–27). PSP is a truncated subtype of soluble CD14, a soluble fragment of the membrane-bound protein cluster of differentiation 14 (CD14) expressed by activated macrophages in response to bacterial lipoglycans (28). Plasma PSP rises significantly in the setting of sepsis, with its concentration proportional to severity (23, 28) and exhibits sensitivity and specificity superior to that of CRP, PCT, and interleukin-6 (22, 23). It has shown diagnostic reliability in many applications, including sepsis, pneumonia, intraabdominal infection, and other extracranial infections (26, 27, 29, 30).

Presepsin can also be detected in the CSF (31) and may indicate microglial activation to bacterial infection within the CNS. A further attractive aspect of measuring CSF PSP is that, as a protein activated by bacterial infections, it should be insensitive to blood in the CSF and chemical meningitis, both common situations that complicate the diagnosis of NI-CNS in neurocritical care patients. A handful of studies have examined PSP in the CSF of children and neonates with bacterial meningitis, with some promising results (31, 32). Its usefulness in adult neurocritical care patients with NI-CNS has yet to be demonstrated. In this study, we aim to establish a normal range for PSP in human CSF and to evaluate for the first time the usefulness of CSF PSP as a marker of NI-CNS.

## Materials and Methods

This prospective observational study, carried out in accordance with Good Clinical Practice and the Declaration of Helsinki, was performed in a tertiary hospital setting. The study was approved by the local ethics committee and participants or their surrogates gave informed, written consent. The study was formed of two parts. The objective of the first component was to establish normal ranges of CSF PSP, while the second was to determine cut-off values predictive of NI-CNS and to provide data on the sensitivity and specificity of CSF PSP for NI-CNS. We also aimed to determine whether the presence of blood in the CSF affects PSP levels.

In the first study component, a control group of adult patients undergoing elective surgery for urological pathology requiring spinal anesthesia were recruited. Exclusion criteria included individuals under 18 years of age, the presence of concomitant neurological or neurosurgical pathology, or refusal to participate in the study. In each patient, a CSF sample was obtained at induction of spinal anesthesia. Contemporaneous blood samples were not taken and only PSP was measured in the CSF.

In the second component, neurocritical care patients aged 18 and older with suspected NI-CNS were recruited. In our institution, patients are considered for neurocritical care if they meet the following criteria: altered consciousness with a Richmond Agitation-Sedation Scale of +3 or +4 or −3 through −5, including those in coma or requiring prolonged sedation; respiratory insufficiency requiring mechanical ventilation; hemodynamic instability requiring inotropic or vasopressor support; or severe electrolyte disturbances (e.g., Na > 165 or <125 mmol/l, K > 6.5 mmol/l) with high risk of complications.

Patients were excluded if they had any contraindication to lumbar puncture (unless they had a ventricular drain). Other exclusion criteria comprised brain death and the refusal by the patient or their surrogates to participate. Patients were treated in accordance with international guidelines (33). In patients with suspected or confirmed NI-CNS, blood and CSF samples were obtained simultaneously whenever clinical indications warranted CSF sampling. CSF analysis included determination of cell count, glucose, lactate, and PSP. CSF was also sent for microbiological culture and pathogen verification. CSF PCR was also performed where possible. Contemporaneous blood sampling comprised white cell count, CRP, PCT, PSP, and glucose.

For this study, NI-CNS was diagnosed when the following criteria were met with or without positive bacterial CSF culture: clinical suspicion [new-onset altered consciousness, reduction in GCS, new-onset seizures (3)], CSF cell count >300/μl (34), CSF glucose:serum ratio <0.4, and CSF lactate >2.1 mmol/l (35, 36). Colonization of intracranial devices was determined when the following criteria were met: absence of clinical suspicion of NI-CNS, CSF analysis not meeting the criteria for NI-CNS, and more than one positive microbiological culture of organisms typically causing NI-CNS. CSF sample contamination was defined as the absence of a clinical picture consistent with NI-CNS, CSF analysis not meeting the criteria for NI-CNS, and a single positive microbiological culture of organisms atypical for NI-CNS, which either yielded different organisms or no growth on subsequent sampling. CSF from the ventricular drain was only sampled in the context of suspected NI-CNS; routine infection screening was not carried out. We did not routinely irrigate our external drainage systems and only disconnected them to change a full drainage bag. Diagnosis of systemic infection (pneumonia, urinary tract infection, sepsis, or surgical site infection) was based on the criteria and guidelines of the Centers for Disease Control and Prevention (37–39).

### Statistical Analysis

Statistical analysis was performed using SPSS version 23.0 (IBM Corp., Armonk, NY, USA). The Shapiro–Wilk method was used to test for distribution normality. All comparisons between groups were carried out using non-parametric tests (Mann–Whitney *U*-test or the Wilcoxon test as appropriate), with statistical significance set at the *p* ≤ 0.05 level. Specificity and sensitivity of CSF PSP in the different patient groups were assessed using the receiver operating characteristic (ROC) toolbox in SPSS.

## Results

Fifteen CSF samples were obtained during routine spinal anesthesia for the determination of normal CSF PSP ranges. The mean age of these control participants was (mean ± SD) 59.3 ± 14.1 years and all were male. None required postoperative ICU admission, and outcomes were favorable in all cases. CSF PSP was 75.32 ± 25.32 pg/ml. Values were normally distributed, allowing us to conclude that the normal range of CSF PSP in patients with systemic infection, brain injury, or NI-CNS is 50–100 pg/ml.

Twenty-one neurocritical care patients with suspected or confirmed NI-CNS were included in the study. Mean age was 50.7 ± 15.0 years. The pathologies leading to ICU admission are listed in Table 1.

**Table 1:** Pathology leading to intensive care unit admission.

Pathology	Number of patients
Brain tumor	6
Intraventricular hemorrhage	6
Traumatic brain injury	4
Ischemic stroke	3
Subarachnoid hemorrhage	1
Polyneuropathy	1

Sixteen patients required mechanical ventilation during their stay (mean duration 11.3 ± 10.0 days), with fifteen undergoing a tracheostomy. The non-intubated patients were admitted either for vasopressor support in the context of hemodynamic instability (4 patients) or for treatment of hyponatremia (1 patient, Na 118 mmol/l). Mean length of stay in ICU was 16.5 ± 9.2 days, with total hospital stay 37.6 ± 37.0 days. NI-CNS was diagnosed in 10 patients. Onset of NI-CNS was 5.9 ± 3.5 days post-operation/ictus. Causative microorganisms were identified in three patients: *Proteus mirabilis* (1), *Streptococcus pneumonia* (1), and *Enterococcus faecium* (1). Risk factors for NI-CNS and outcomes are presented in Table 2. CSF cell counts, glucose and lactate, and systemic inflammation markers (CRP, PCT, leukocytes) are presented in Table 3. No instances of device or sample contamination were detected in this cohort.

**Table 2:** Risk factors for NI-CNS and outcomes in patients with (NI-CNS+) and without (NI-CNS−) nosocomial infection of the central nervous system.

	Number of patients with EVD and total duration	Number of patients with LD and total duration	Number of patients with no indwelling device	CSF leak	Skull base fracture	Intraventricular hemorrhage	Subarachnoid hemorrhage	Neurosurgical operations (other than EVD)	GOS		
									3–5	1–2	1
NI-CNS+	5 (8.6 ± 3.1)	1 (4)	7	1	–	4	1	9	9	–	2
NI-CNS−	1 (12)	1 (3)	6	1	1	2	–	3	4	1	5

*EVD, external ventricular drain; LD, lumbar drain; GOS, Glasgow Outcome Scale*.

**Table 3:** Cerebrospinal fluid (CSF) and systemic markers of inflammation in patients with (NI-CNS+) and without (NI-CNS−) nosocomial infection of the central nervous system.

	CSF cell count	CSF glucose	CSF lactate	CRP	PCT	Leukocytes
NI-CNS+	486.6 ± 699.6	3.95 ± 1.83	7.0 ± 14.7	84.4 ± 69.9	0.66 ± 0.92	11.7 ± 3.7
NI-CNS−	51.2 ± 95.6	5.0 ± 1.4	3.5 ± 1.1	85.7 ± 64.9	3 ± 4.2	14.1 ± 6.5

*CRP, C-reactive protein; PCT, procalcitonin*.

In total, 97 pairs of CSF and blood samples were obtained. All pairs were divided into four groups defined by the presence or absence of NI-CNS and/or systemic infection (Table 4). The distribution of values was normal in all groups.

**Table 4:** Distribution of cerebrospinal fluid and blood samples.

		NI-CNS	
		Yes	No
Systemic infection	Yes	22 (SI+, NI-CNS+)	32 (SI+, NI-CNS−)
	No	21 (SI−, NI-CNS+)	22 (SI−, NI-CNS−)

*NI-CNS, nosocomial infection of the central nervous system*.

**Figure 1:**
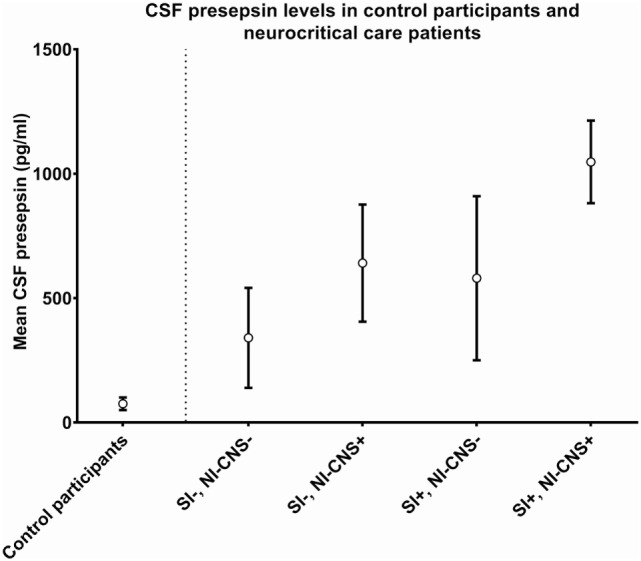
Cerebrospinal fluid (CSF) values for (left) control participants undergoing routine spinal anesthesia and (right) neurocritical care patients with and without systemic and/or nosocomial infection of the CNS. SI−, no systemic infection; SI+, systemic infection; NI-CNS−, no nosocomial infection of the CNS; NI-CNS+, nosocomial infection of the CNS. Error bars represent the SD.

**Figure 2:**
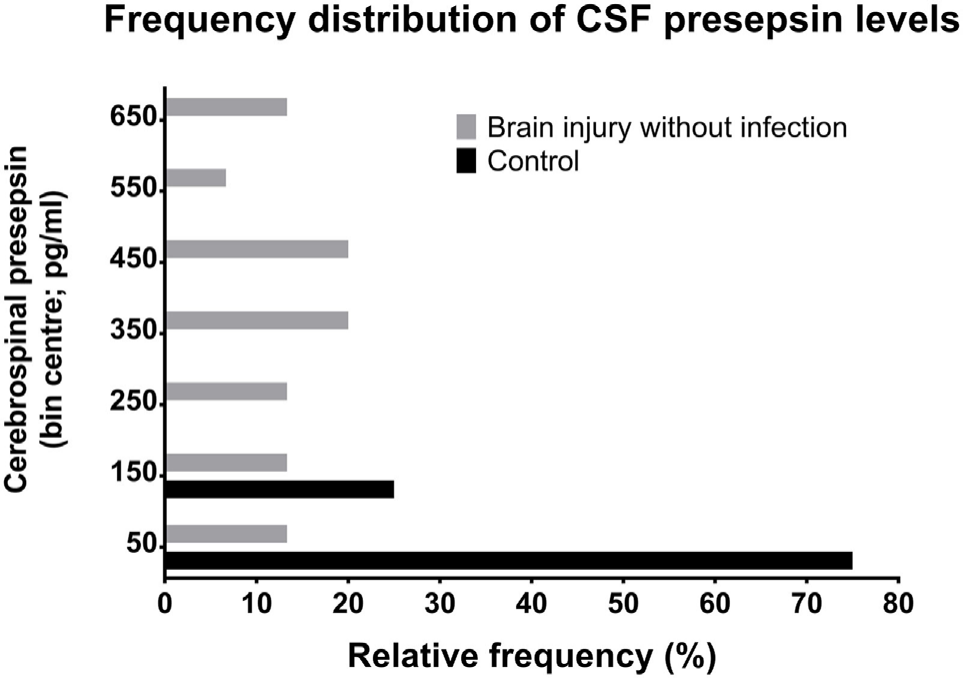
Relative frequency distributions of CSF PSP values in non-neurocritical care patients and in neurocritical care patients without NI-CNS. PSP, presepsin; NI-CNS, nosocomial infection of the CNS; CSF, cerebrospinal fluid.

In cases with neither NI-CNS nor systemic infection (SI−, NI-CNS−), CSF PSP was 340.4 ± 201.1 pg/ml. This level of CSF PSP, obtained from neurocritical care patients, is significantly higher than that found in the CSF of the control participants investigated during the first part of the study (75.32 ± 25.32 pg/ml; Figures 1 and 2). In patients with systemic infection but without NI-CNS (SI+, NI-CNS−), CSF PSP was 580.1 ± 329.7 pg/ml. In patients with NI-CNS but without systemic infection (SI−, NI-CNS+), CSF PSP was 640.8 ± 235.5 pg/ml, twice as high as neurocritical care patients with neither systemic nor CNS infection. In cases with both NI-CNS and systemic infection (SI+, NI-CNS+), CSF PSP was 1,047.7 ± 166.2 pg/ml.

**Figure 3:**
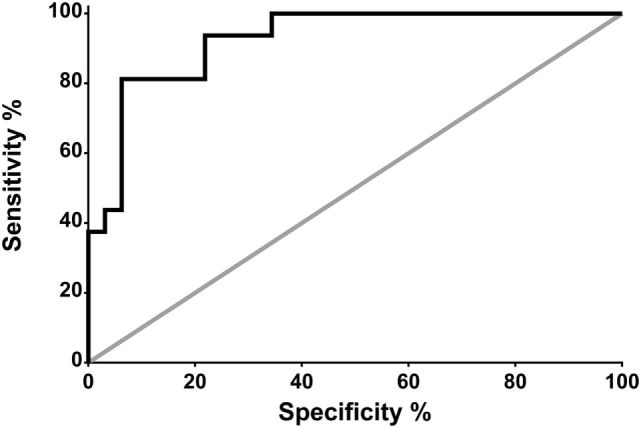
Receiver operating characteristic (ROC) analysis: prediction of NS-CNS+ status using CSF PSP in neurocritical care patients without systemic infection (groups SI−, NI-CNS− and SI−, NI-CNS+). NI-CNS−, no nosocomial infection of the central nervous system; NI-CNS+, nosocomial infection of the CNS; CSF, cerebrospinal fluid; PSP, presepsin.

Receiver operating characteristic analysis revealed that, in neurocritical care patients without systemic infection, a CSF PSP of 321 pg/ml is associated with a sensitivity and specificity for NI-CNS of 100 and 58.3%, respectively (Figure 3).

**Figure 4:**
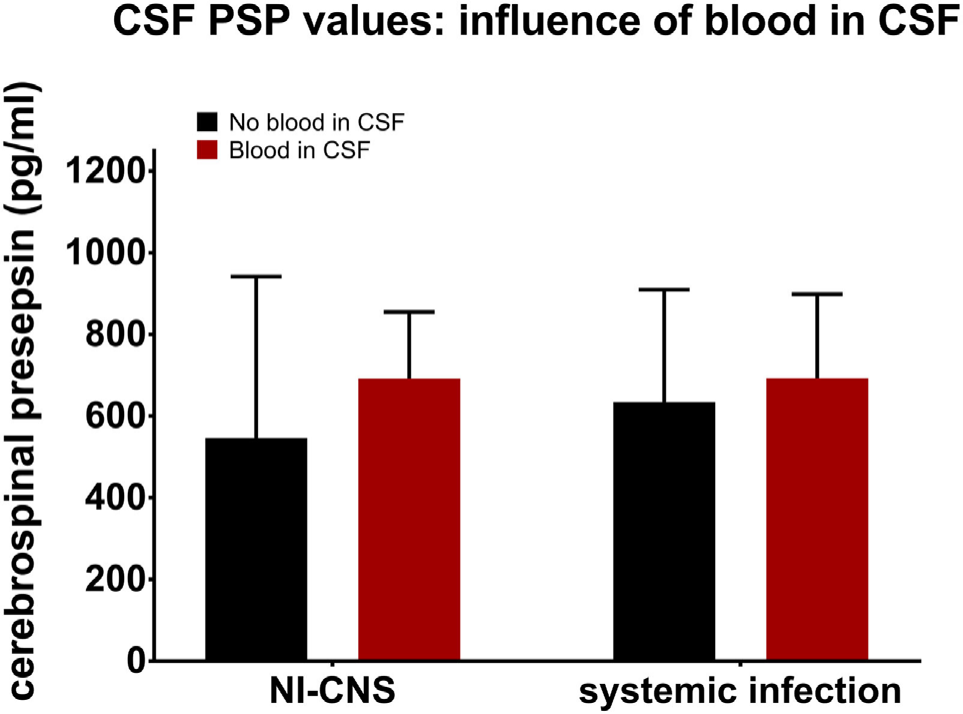
Influence of CSF blood on CSF PSP values. NI-CNS, nosocomial infection of the central nervous system; CSF, cerebrospinal fluid; PSP, presepsin.

Presence or absence of blood in the CSF was determined and then analyzed to assess its influence on CSF PSP levels. This analysis was performed in samples with NI-CNS, but without systemic infection and those without NI-CNS, but with systemic infection. Statistical analysis revealed that the presence of blood did not influence the level of CSF PSP in either group (SI−, NI-CNS+; *p* = 0.144, SI+, NI-CNS−; *p* = 1.00, Mann–Whitney *U*-test; Figure 4).

## Discussion

To the best of our knowledge, this is the first study to investigate the utility of CSF PSP in the diagnosis of NI-CNS in adult neurocritical care patients. We report normal values for CSF PSP in adults without brain injury or infection of 50–100 pg/ml. CSF PSP levels are significantly elevated in neurocritical care patients and rise still further in the presence of NI-CNS and systemic infection. Very high levels of CSF PSP (>800 ng/ml) are seen in the context of simultaneous NI-CNS and systemic infection. Finally, the presence of blood in the CSF does not appear to significantly influence CSF PSP levels.

The diagnosis of NI-CNS in contemporary neurocritical care is frequently presumptive due to the low sensitivity and specificity of traditional markers, a fact well demonstrated in our cohort (Table 3). New, reliable biomarkers are urgently required to assist early diagnosis and subsequent monitoring of therapy in NS-CNS. A soluble, truncated protein fragment of the macrophage surface protein CD14, PSP is a hypothetically appealing CNS infection marker as it is produced in the systemic circulation by macrophages, and thus likely secreted by CNS microglia, in response to bacterial infection (25, 31, 40, 41). PSP has been successfully introduced into general critical care practice as a novel biomarker for bacterial inflammation (22–24) and has shown promise as a CSF infection marker in neonates (31).

Almost nothing is known regarding PSP behavior in the CSF of adult neurocritical care patients, with or without NI-CNS. It was therefore necessary to establish a normal range using an appropriate control population without brain injury. In our cohort of urological patients undergoing routine spinal anesthesia, the normal range of CSF PSP was 50–100 pg/ml. Certain caveats pertain to this finding; this small control group consisted only of older males prior to undergoing surgery for urological tumors. These factors (age, gender, and suspected urological malignancy) may influence CSF PSP and care should be exercised in extrapolating these values to other groups. For comparison, 123 control participants had a median serum PSP of 123 pg/ml (IQR: 89–155 pg/ml) (42), and thus we believe these data to be reflective of the general population without neurological injury or infection. Only PSP levels were measured in the CSF of the control population, meaning that correlations with other established markers of CNS inflammation, such as cell count, glucose, and protein, were not possible.

Neurocritical care patients invariably have some degree of brain injury (43). Thus, in keeping with evidence that microglia respond to any type of neurological injury (44), CSF PSP is likely to be elevated in these patients. Our group of patients with neither NI-CNS nor systemic infection (SI−, NI-CNS−) reflects the clinical scenario of isolated, non-infectious brain injury. These patients indeed had CSF PSP levels of 304 ± 201.1 pg/ml, significantly higher than that of the control group and supporting this microglial activation hypothesis.

Systemic infections have considerable effects on neurological function (45). Delirium develops in up to 82% of ICU patients, and systemic infection is a principal risk factor (46). The septic state is often associated with neurotoxicity and encephalopathy (45, 47) and associated microglial activation (48). Our data are in line with this perspective; systemic infection in the absence of NI-CNS was associated with a CSF PSP of 580.1 ± 329.7 pg/ml, markedly higher than levels in the CSF of control subjects, but not significantly different from samples taken from neurocritical care patients with neither NI-CNS or systemic infection nor with isolated NI-CNS.

Infection of the CNS stimulates an immune response and microglia activation. It may be expected that this response would be more pronounced in NI-CNS than in brain injury without infection or in neurocritical care patients with systemic infection only. These data indicate that CSF PSP levels in such cases (640 ± 235.5 pg/ml) are indeed higher than the other two groups, but not significantly so.

The highest level of CSF PSP was observed in patients with neurological injury with both NI-CNS and systemic infection, with levels frequently over 1,000 pg/ml.

Receiver operating characteristic analysis of these preliminary data indicates that a CSF PSP cut-off value of 321 pg/ml and above has a sensitivity of 100% and a specificity of 58.3% for NI-CNS in neurocritical care patients without systemic infection. The relatively low specificity was in keeping with the high SD observed in these two groups of patients, with the optimal cut-off value falling below the mean of the group without NI-CNS (340 ng/ml; Figure 1). It is important to note that these results must be interpreted in the absence of a true “gold standard” in the diagnosis of NI-CNS, as false positives and negatives are likely despite use of evidence-based guidelines in the diagnosis of our cohort. While PCR analyses may accelerate and increase the sensitivity of diagnosis (49), distinguishing infection from contamination remains challenging.

Taken collectively, these data provide grounds to use CSF PSP in the diagnosis of NI-CNS only as an additional marker. Indeed, CSF PSP discriminated particularly poorly between our patients with systemic infection and those with isolated nosocomial CNS infection. However, there is reason to be optimistic that it may be possible to define sensitive and specific cut-off values for CSF PSP for particular clinical situations such as isolated brain injury, intraventricular hemorrhage, CNS infection, and so on, as is the case with PCT (16, 50). Combination with other markers, perhaps those more sensitive to systemic infection, may be a productive approach (51). The ALBIOS trial, a multicenter, randomized trial of PSP in patients with severe sepsis suggested that serum PSP may play a key role in identifying those patients who do not respond adequately to therapy, potentially due to inappropriate antibiotic therapy (27, 52). A similar role in nosocomial CNS infection, in which clinical or biochemical monitoring of treatment is often particularly challenging, would be a useful addition to the intensivist’s armory. To this end, further research of CSF PSP in neurocritical care patients seems entirely warranted.

Blood in the CSF complicates the diagnosis of NI-CNS for several reasons. First, cell counts are always raised in subarachnoid hemorrhage and postoperative patients and, while centrifugation of the CSF sample may aid in discrimination between a postictal state and CNS infection, this is not always reliable (53). Second, blood cells may consume glucose and produce lactate, mimicking bacterial CNS infection (54). Finally, neurosurgical interventions, including many treatments for subarachnoid and intraventricular hemorrhage, are risk factors for CNS infection (55). These facts highlight the need for a marker of CNS infection which is not influenced by blood in the CSF. Our data suggest this to be the case for CSF PSP.

This preliminary, prospective analysis is subject to the limitations inherent to such pilot studies. The number of patients and samples were small and from a single institution and, while all data were normally distributed and patients were representative of the target group in which PSP may be used, the cut-off value indicated by the ROC analysis should be interpreted with caution.

## Conclusion

Cerebrospinal fluid PSP holds potential as a diagnostic marker for NI-CNS. The normal level of PSP in the CSF is 50–100 pg/ml, whereas a CSF PSP of more than 321 pg/ml in neurocritical care patients without systemic infection is associated with 100% sensitivity and 58.3% specificity for NI-CNS. Currently, the role of CSF PSP in diagnosing NI-CNS is limited to application as an additional marker, aiding the interpretation of the clinical picture, including other routine infection parameters.

## Ethics Statement

All procedures performed in studies involving human participants were in accordance with the ethical standards of the 1964 Helsinki declaration and its later amendments. The study was approved by the research ethics committee of the Burnazian State Research Center. Informed consent was obtained from all individual participants included in the study.

## Author Contributions

NK, KB, IL, MZ, AS and EC designed and carried out the study, analyzed and interpreted data and reviewed the manuscript. SA designed and carried out the study, analyzed and interpreted data and wrote the manuscript. KK and TK performed statistical analysis, analyzed and interpreted data and reviewed wrote the manuscript. OP and YU analyzed and interpreted data and reviewed the manuscript. KP conceived and acted as sponsor and guarantor of the study, designed and carried out the study, analyzed and interpreted data and wrote the manuscript.

## Conflict of Interest Statement

The authors declare that the research was conducted in the absence of any commercial or financial relationships that could be construed as a potential conflict of interest.

## Abbreviations

CD14, cluster of differentiation 14; sCD14, soluble CD14; sCD14-ST, soluble CD14 subtype (presepsin); CNS, central nervous system; CRP, C-reactive protein; CSF, cerebrospinal fluid; EVD, external ventricular drainage; GOS, Glasgow Outcome Scale; ICU, intensive care unit; IL-6, interleukin 6; LD, lumbar drainage; NI-CNS, nosocomial infection of the CNS; PCR, polymerase chain reaction; PCT, procalcitonin; PSP, presepsin; RASS, Richmond Agitation-Sedation Scale; ROC, receiver operating characteristic; SI−, systemic infection absent; SI+, systemic infection present.
